# Experimenting with modifications to consent forms in comparative effectiveness research: understanding the impact of language about financial implications and key information

**DOI:** 10.1186/s12910-021-00736-x

**Published:** 2022-03-27

**Authors:** Nyiramugisha K. Niyibizi, Candace D. Speight, Gabriel Najarro, Andrea R. Mitchell, Ofer Sadan, Yi-An Ko, Neal W. Dickert

**Affiliations:** 1 Georgia Clinical and Translational Science Alliance (Georgia CTSA), Emory University School of Medicine, 1462 Clifton Rd, #526, Atlanta, GA 30322 USA; 2 Division of Cardiology, Department of Medicine, Emory University School of Medicine, Atlanta, GA USA; 3 Department of Neurology and Neurosurgery, Emory University School of Medicine, Atlanta, GA USA; 4 Department of Biostatistics and Bioinformatics, Emory University Rollins School of Public Health, Atlanta, Georgia USA

**Keywords:** Informed consent, Compensation for injury, Key information, Consent language

## Abstract

**Background:**

Informed consent forms are intended to facilitate research enrollment decisions. However, the technical language in institutional templates can be unfamiliar and confusing for decision-makers. Standardized language describing financial implications of participation, namely compensation for injury and costs of care associated with participating, can be complex and could be a deterrent for potential participants. This standardized language may also be misleading in the context of comparative effectiveness trials of standard care interventions, in which costs and risk of injury associated with participating may not differ from regular medical care. In addition, the revised U.S. Common Rule contains a new requirement to present key information upfront; the impact of how this requirement is operationalized on comprehension and likelihood of enrollment for a given study is unknown.

**Methods:**

Two online surveys assessed the impact of (1) changes to compensation for injury language (standard vs. tailored language form) and (2) changes to the key information page (using the tailored compensation language form with standard key information vs. modified key information vs. modified key information plus financial information) on both likelihood of enrollment in and understanding of a hypothetical comparative effectiveness trial.

**Results:**

Likelihood of enrolling was not observed to be different between the standard and tailored language forms in Study 1 (73 vs. 75%; *p* = 0.6); however, the tailored language group had a higher frequency of understanding the compensation for injury process specific to the trial (25 vs. 51%; *p* < 0.0001). Modifications to the key information sheet in Study 2 did not affect likelihood of enrolling (88 vs. 85 vs. 85%; *p* = 0.6); however, understanding of randomization differed by form (44 vs. 59 vs. 46%; *p* = 0.002).

**Conclusions:**

These findings suggest that refining consent forms to clarify key information and tailoring compensation for injury language to the nature of the study, especially in the context of comparative effectiveness trials, may help to improve study comprehension but may not impact enrollment.

**Supplementary Information:**

The online version contains supplementary material available at 10.1186/s12910-021-00736-x.

## Background

Defining an optimal approach to informed consent for comparative effectiveness trials—clinical trials comparing interventions delivered as part of standard practice—has been controversial. There has been ongoing debate regarding how risks are categorized, for example, and when consent is necessary [1–3]. Part of the latter discussion is how informed consent forms (ICF) should be constructed and what information should be presented. Although informed consent is a process that cannot be reduced to a form, ICFs are the face of the consent process during Institutional Review Board (IRB) review, and they represent the standardized presentation of information that all potential participants receive. Their impact on potential participants’ understanding and enrollment decisions is important to study, and data regarding possible adaptations or alterations to ICFs in comparative effectiveness trials may help to inform practice.

One area in which comparative effectiveness trials may differ from trials of novel interventions is potential financial implications of participation, especially in the event of harm or injury. When all arms of a trial represent standard therapy, any complications related to study treatment are not discrete from complications of standard clinical care. However, our experience in the context of a recent trial in neurocritical care suggested that compensation for injury and costs of care (insurance coverage) language within a standard institutional template—which highlights potential differences with regard to care for complications from research interventions—may have driven individuals to decline participation in a comparative effectiveness trial comparing two standard treatments in subarachnoid hemorrhage [4]. In addition, in a study collaborating with patient and surrogate advisors to design consent forms and processes for other trials, our advisors consistently mentioned that these issues mattered to them and that institutional template language is both confusing and off-putting [5]. The impact of this language on actual decision-making is uncertain.

An additional challenge in constructing ICFs for comparative effectiveness studies in the United States is how “key information” should be presented. The recent revision to the Common Rule introduced a requirement for a concise presentation of “key information” at the beginning of an ICF that is most relevant for potential participants to consider [6]. This requirement states that ICFs should highlight information that matters to participants, but little guidance exists for investigators or IRBs regarding what information to include or how to present it. In the context of comparative effectiveness trials, it is unclear how much to emphasize the fact that all interventions are part of standard of care. There may be worries that over-emphasizing this aspect of the study could obscure appreciation of research risks and benefits, for example. Because this section may be part of developing potential participants’ “first impression” of a study, it is important to assess the ways in which it might structure potential participants’ thoughts about and attitudes toward a study.

To address the challenge of standardized, templated language, there have been efforts to make consent forms more accessible and concise [7–12], but the impact of such changes remain uncertain, and the specific impact of different approaches to these two portions of ICFs in the context of comparative effectiveness research is unknown. In order to investigate the potential impact of changes in language regarding financial implications and to increase understanding of the impact of different constructions of the “key information” section, we conducted a series of online survey experiments that compared hypothetical willingness to enroll in a clinical trial when presented with modified versions of ICFs.

## Methods

The objective of this study was to assess the impact of two sets of modifications to the ICF on hypothetical willingness to enroll in a comparative effectiveness study in neurocritical care. The first modification involved a clearer description of the compensation for injury and insurance coverage sections with language specifically tailored to the comparative effectiveness study. The second set of modifications involved the “key information” section. A simplified, more positively-framed version of the key information section was created with the intention of clarifying key aspects of the study. An additional version of the modified key information section was also created that added a single line about costs involved with participation. The study was conducted online as two sequential experiments (described below). The study was considered exempt from review by the Emory University IRB.

Participants were surveyed using the Amazon Mechanical Turks (MTurk) platform, managed through CloudResearch, between June and August 2020 (www.cloudresearch.com) [13]. MTurk is an online crowdsourcing platform that aids researchers in completing a variety of human intelligence tasks (HITs), including surveys [14]. The study population was made up of members of the general public who were registered with MTurk, and the surveyed population was limited to MTurk members who had earned at least a 98% approval rating.

For both experiments, participants were instructed to read a consent form for a comparative effectiveness trial examining two standard intravenous hypertonic fluids to treat subarachnoid hemorrhage (SAH). They were asked to imagine that they are the medical decision-maker being asked to enroll an incapacitated family member diagnosed with SAH in the study. The consent form (Additional file 1) describing the theoretical study was created using standard consent language from an existing clinical trial that was made more concise and then further revised for simplicity by a patient advisory panel. This revised form was treated as the control (Form A—Standard).

In experiment 1, participants received either Form A (Standard) or Form B (Tailored Compensation Language), which were identical except for modifications to the compensation for injury/insurance coverage sections. Specifically, Form B tailored language in that section to the nature of the trial by emphasizing that none of the treatments were experimental or outside of standard practice and that treatment for research-related injury would be handled just like regular medical care. Participants were randomly assigned in a 1:1 ratio to receive either Form A or Form B.

In experiment 2, Form B was compared against two forms that contained Form B’s changes along with modifications to the concise, key information page (Additional file 2). Form C (Modified Key Information) contained a simplified and more positively-framed key information page. Form D (Clarified Costs) was identical to Form C but specifically added (on the key information page) that there were no extra costs associated with participation, with the intent to address concerns about costs associated with participating. Participants were randomly assigned in a 1:1:1 ratio to receive either Form B, Form C, or Form D. Participants in the first experiment were excluded from participating in the second experiment.

An identical survey instrument was used for both experiments. Major survey domains included understanding of the trial (including the study purpose, randomization to treatment groups, and information regarding compensation for injury), willingness to participate, concerns about participating, and demographic information (Survey available as Additional file 3). In order to ensure quality responses, two attention checking questions were added to evaluate whether participants paid attention to the survey and the consent form. Survey respondents who did not correctly answer both attention checks were excluded from the analysis.

Before the first experiment, the survey instrument underwent 4 rounds of pretesting with 50 participants from MTurks using the standard (Form A) versus tailored compensation language consent form (Form B) and the pretest survey that included options for respondents to indicate areas of confusion in the forms and survey questions. Minor modifications to clarify survey questions were made based on pretest results.

The primary outcome of both studies was willingness to enroll a family member in the trial, as assessed by the question “After reading this consent form how likely are you to give permission to include your family member in this study?” Responses were assessed using a 4-point Likert scale ranging from Very Unlikely to Very Likely, and responses were dichotomized to Unlikely and Likely for the primary analysis. No ‘undecided’ or ‘unsure’ option was included due to the need to collect a more definitive enrollment decision, and initial sample size calculations were based on a dichotomous outcome.

The secondary outcome, understanding of the compensation for injury process, was assessed using the following question: “If you are injured or harmed as a result of being in this study, how will your care be paid for?” Understanding of the study itself was assessed by asking, “Which of the following best describes how treatment will be decided for patients in this study?” and “What best describes what this study is testing?”.

The two experiments were conducted sequentially and analyzed separately. In experiment 1, a sample size of 650 (325 per group) was estimated to provide 80% power with a two-sided alpha level of 0.05 to detect a 10% difference in hypothetical willingness to enroll in the trial between the two arms. Using the baseline willingness to enroll in the study observed in experiment 1, a sample size of 750 (250 per group) was estimated for Experiment 2 to provide 80% power with a two-sided alpha level of 0.05 to detect a 10% difference in hypothetical willingness to enroll in the trial.

Analyses were conducted using SAS 9.4 (Cary, NC). Descriptive statistics were used to summarize respondent demographic characteristics and survey responses across consent form groups, including the primary outcome of willingness to enroll a family member in the study. The primary analysis in each experiment involved pairwise comparisons of willingness to enroll between arms using Chi-square tests. Multiple logistic regression was used to examine associations between age, gender, race, education, consent form version, and understanding of the compensation for injury process with hypothetical willingness to enroll a family member in the trial. The analysis plan for both experiments was pre-registered with AsPredicted (www.aspredicted.org/ #44180).

## Results

### Experiment 1: compensation for injury language

Overall, 776 respondents completed the survey, of which 118 failed the attention check questions. Among 658 quality responses, 319 received the standard form (Form A), and 339 received the tailored compensation language form (Form B). Participant demographic characteristics were balanced across groups (Table 1). Participants had higher educational attainment than the general US population.

**Table 1:** Demographics by consent form versions

	Experiment 1		Experiment 2		
	Form A: standard (n = 319) N (%)	Form B: tailored compensation (n = 339) N (%)	Form B: tailored compensation/standard key information (n = 248) N (%)	Form C: modified key information (n = 254) N (%)	Form D: clarified costs (n = 251) N (%)
Age (years)					
18–29	77 (24.1)	70 (20.7)	56 (22.6)	54 (21.3)	58 (23.1)
30–44	157 (49.2)	166 (49.0)	141 (56.9)	145 (57.1)	132 (52.6)
45–59	70 (21.9)	74 (21.8)	36 (14.5)	46 (18.1)	48 (19.1)
60+	15 (4.7)	26 (7.7)	14 (5.7)	8 (3.2)	13 (5.2)
Gender					
Male	202 (63.3)	197 (58.1)	161 (64.9)	161 (63.4)	143 (57.0)
Female	116 (36.4)	136 (40.1)	85 (34.3)	91 (35.8)	106 (42.2)
Prefer to self-describe/prefer not to answer	1 (0.3)	6 (1.8)	2 (0.8)	2 (0.8)	2 (0.8)
Race					
Asian	18 (5.6)	18 (5.3)	6 (2.4)	14 (5.5)	9 (3.6)
Black or African American	31 (9.7)	46 (13.6)	50 (20.2)	43 (16.9)	44 (17.5)
Hispanic/Latino(a)	21 (6.6)	11 (3.2)	14 (5.6)	9 (3.5)	9 (3.6)
White or European American	239 (74.9)	254 (74.9)	164 (66.1)	175 (68.9)	178 (70.9)
Other	6 (1.9)	5 (1.5)	13 (5.2)	9 (3.5)	8 (3.2)
Education					
Less than high school	0	1 (0.3)	0	1 (0.1)	0
High school graduate or GED	37 (11.6)	35 (10.3)	15 (6.0)	54 (7.2)	15 (6.0)
Some college	79 (24.8)	79 (23.3)	42 (16.7)	121 (16.1)	42 (16.7)
Bachelor’s degree	157 (49.2)	176 (51.9)	140 (55.8)	435 (57.8)	140 (55.8)
Postgraduate or Professional degree	46 (14.4)	48 (14.2)	54 (21.5)	142 (18.9)	54 (21.5)

Demographic characteristics of survey respondents by experiment and consent form version

**Fig. 1:**
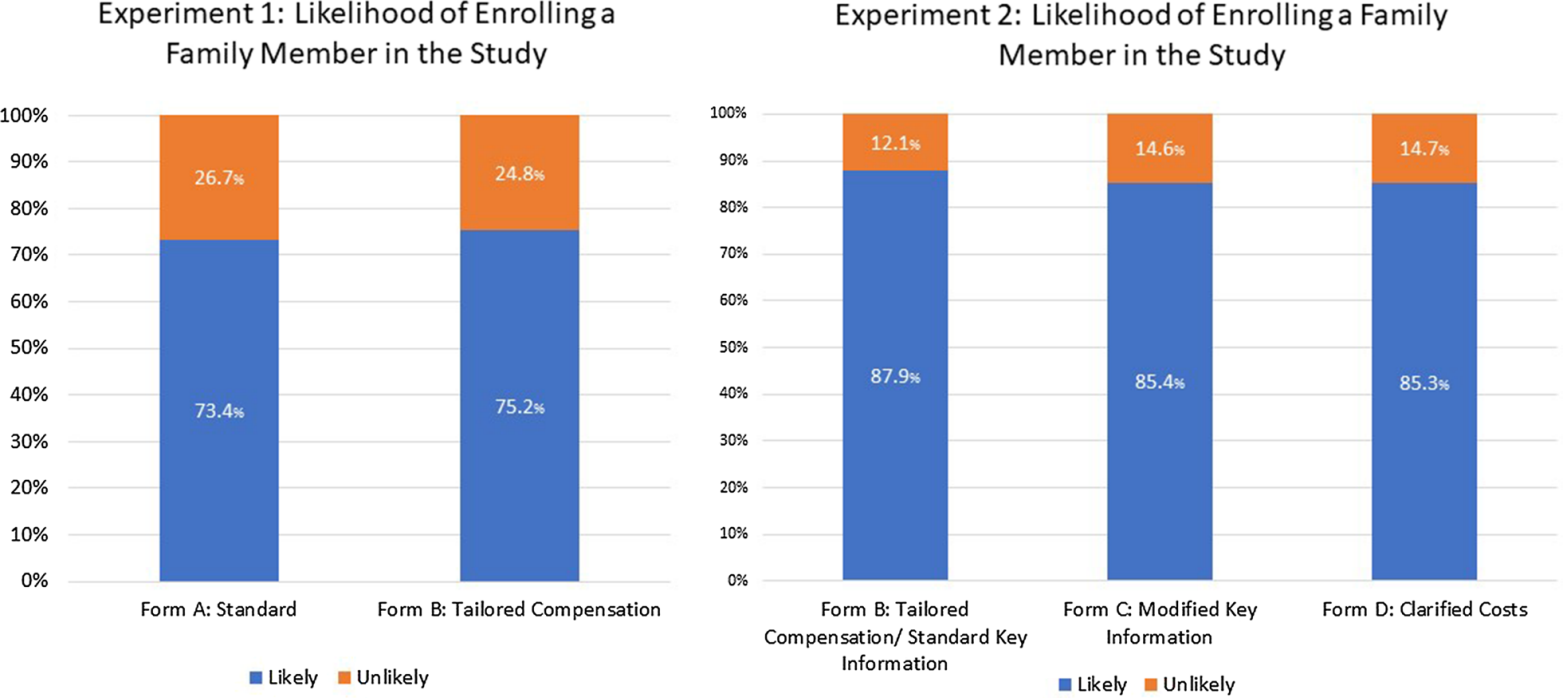
Likelihood of enrolling a family member in the study by consent form—experiments 1 and 2. Percentage of participants who indicated they were likely or unlikely to enroll a family member into the hypothetical study by consent form version in both experiments

Across both groups, 74% of participants responded that they would likely enroll a family member in the study, and there were no significant differences in willingness to enroll between those who viewed the standard (Form A) and those who viewed the tailored (Form B) compensation language (73% vs. 75%, *p* = 0.5838) (Fig. 1). Overall, the most common concerns about enrollment were about risk (68%), costs (48%), and getting the less effective treatment (47%) (Additional file 4). Frequencies of these 3 concerns did not vary significantly by consent form group.

Individuals assigned to Form B were more likely to answer that injuries would be handled the same as regular medical care (51 vs. 25%, *p* < 0.0001) and to be confident in their answer to this question (83 vs. 68%, *p* = 0.02) (Table 2). More participants who viewed the standard form (Form A) selected the response that insurance companies may treat research harms differently than regular medical care (32 vs. 12%, *p* < 0.0001). Across both form groups, the most incorrect answer—that the sponsor would provide compensation free of charge—was associated with a higher willingness to participate than the answer that injuries would be handled the same as regular medical care (87% vs. 73%, *p* = 0.01).

**Table 2:** Compensation for injury by consent form

If you are injured or harmed as a result of being in this study, how will your care be paid for?	Experiment 1		Experiment 2		
	Form A: standard (n = 319) N (%)	Form B: tailored compensation (n = 339) N (%)	Form B: tailored compensation/standard key information (n = 248) N (%)	Form C: modified key information (n = 254) N (%)	Form D: clarified costs (n = 251) N (%)
Insurance companies may treat research harms or injuries differently than regular medical care.	101 (31.6)	40 (11.8)	46 (18.6)	38 (15.0)	54 (21.5)
It is not clear based on the information I read.	54 (16.9)	62 (18.3)	26 (10.5)	33 (13.0)	19 (7.6)
Just like medical care outside of a research study.	80 (25.1)	173 (51.0)	106 (42.7)	126 (49.6)	109 (43.4)
It may be different for every patient.	39 (12.2)	26 (7.7)	35 (14.1)	29 (11.4)	28 (11.2)
The sponsor of the study will provide it free of charge.	45 (14.1)	38 (11.2)	35 (14.1)	28 (11.0)	41 (16.3)

Responses to compensation for injury language survey question. ‘Just like medical care outside of a research study’ was intended to be the most accurate response for the nature of the study

In a multiple logistic regression model, there was no significant impact of consent form assignment on likelihood of enrolling in the study (Table 3). Asian race was associated with lower odds of enrolling in the study compared to Whites [OR = 0.38 (0.18, 0.79)], and having at least a Bachelor’s degree was associated with higher odds of enrolling in the study [OR = 1.87 (1.29, 2.71)] compared to some college or less. There was no significant interaction between response to the compensation for injury question and consent form version regarding likelihood of enrolling when an interaction term was added to this model.

**Table 3:** The association between consent form and likelihood of enrollment with demographic covariates, experiment 1

Characteristics	OR	95% CI		*p* value
Form	0.911	0.633	1.31	0.615
Form A: standard versus Form B: tailored compensation				
Age (year)	0.995	0.98	1.011	0.577
Race/ethnicity				
Black versus White	2.054	0.976	4.321	0.058
Asian versus White	0.381	0.184	0.791	0.010
Hispanic/Latino versus White	0.912	0.403	2.061	0.824
Other versus White	0.559	0.213	1.47	0.239
Sex				
Female versus Male	0.728	0.501	1.058	0.097
Education				
Bachelor’s degree or more versus Some college or less	1.868	1.288	2.711	0.001

Logistic regression model examining the association between consent form version and likelihood of enrollment with demographic covariates in Experiment 1 (comparison of standard vs. tailored compensation for injury language)

Understanding of the clinical trial itself was similar across the two forms (Table 4). Overall, 79% of participants correctly answered “What best describes what this study is testing”, and 68% of participants correctly answered “Which of the following best describes how treatment will be decided for patients in this study?” As with misunderstanding of compensation for injury, participants who answered both questions incorrectly regarding the study itself had higher willingness to enroll a family member in the study compared to those who answered both questions correctly (94 vs. 65%, *p* < 0.0001).

**Table 4:** Understanding of the study by consent form

	Experiment 1		Experiment 2		
	Form A: standard (n = 319) N (%)	Form B: tailored compensation (n = 339) N (%)	Form B: tailored compensation/standard key information (n = 248) N (%)	Form C: modified key information (n = 254) N (%)	Form D: clarified costs (n = 251) N (%)
*Which of the following best describes how treatment will be decided for patients in this study?*					
A computer will randomly assign each patient to one of the two treatment groups. *(correct)*	218 (68.3)	231 (68.1)	109 (43.9)	149 (58.66)	116 (46.22)
A doctor will decide each patient’s treatment group based on what he/she thinks is best for that patient.	70 (21.9)	79 (23.3)	116 (46.8)	85 (33.46)	120 (47.81)
The patient’s family member will decide which treatment the patient receives.	31 (9.7)	29 (8.6)	23 (9.3)	20 (7.87)	15 (5.98)
*What best describes what this study is testing?*					
How well patients with this type of stroke do when treated with one type of IV fluid compared to another. *(correct)*	254 (79.6)	268 (79.0)	169 (68.2)	181 (71.26)	178 (70.92)
The frequency of different complications with this type of stroke based on where the patient is treated.	16 (5.0)	30 (8.9)	28 (11.3)	25 (9.84)	32 (12.75)
The impact of patients’ blood types on rates of recovery from this type of stroke.	49 (15.4)	41 (12.1)	51 (20.6)	48 (18.90)	41 (16.33)
*Both incorrect*	44 (13.8)	44 (13.0)	69 (27.8)	55 (21.7)	62 (33.3)
*One correct*	78 (24.5)	91 (26.8)	80 (32.3)	68 (29.3)	84 (36.2)
*Both correct*	197 (61.8)	204 (60.2)	99 (39.9)	131 (39.1)	105 (31.3)

Responses to survey questions indicating understanding of the hypothetical study by experiment and consent form version

### Experiment 2: modifying key information

957 respondents completed the second survey, with 204 failing the attention check questions. Among 753 quality responses, 248 received the tailored compensation language/standard key information form (Form B), 254 received the modified key information form (Form C), and 251 received the clarified costs form (Form D). Demographic characteristics were balanced across the groups (Table 1). Participants had a higher level of educational attainment than in Experiment 1, with 77% of participants having attained at least a Bachelor’s degree (77 vs. 65%). The population of Experiment 2 also had a higher percentage of Black participants (18 vs. 12%), and a higher percentage of people who had previous experience as a medical decision-maker (55 vs. 34%).

There was no significant difference across the three consent form groups in Experiment 2 regarding the likelihood of enrolling a family member in the study (standard key information: 88%, modified key information: 85%, and clarified costs: 85%; *p* = 0.6) (Fig. 1). The most common concerns overall in Experiment 2 were about risk (57%), costs (54%), and privacy of health information (44%); frequencies of these 3 concerns did not vary significantly by consent form group (Additional file 5).

Understanding of compensation for injury (Table 2) was not significantly affected by consent form assignment in Experiment 2, with no more than 50% of participants answering that injuries would be treated the same as regular medical care in any consent form group (standard key information: 43%, modified key information: 50%, clarified costs: 43%, *p* = 0.2). The highest likelihood of enrollment was observed among those who selected the most incorrect response (‘The sponsor of the study will provide it free of charge’) across all forms (96%).

In a multiple logistic regression analysis, consent form assignment was not significantly associated with willingness to enroll in the study (Table 5). There was also no evidence of an interaction between understanding of compensation for injury and the consent form version on likelihood of enrollment. As in Experiment 1, Asian race was significantly associated with lower odds of enrolling in the study [OR = 0.22 (0.10, 0.51)], and having at least a Bachelor’s degree was associated with higher odds of enrolling in the study [OR = 3.75 (2.38, 5.89)].

**Table 5:** The Association Between Consent Form and Likelihood of Enrollment with Demographic Covariates, Experiment 2

Characteristics	OR	95% CI		*p* value
Form				
Form C: modified key information versus Form B: tailored compensation**/**standard key information	0.932	0.542	1.604	0.800
Form D: clarified costs versus Form B: tailored compensation/standard key information	0.832	0.484	1.428	0.504
Age (year)	1.004	0.984	1.026	0.681
Race/ethnicity				
Black versus White	1.723	0.850	3.496	0.132
Asian versus White	0.221	0.096	0.509	< 0.0001
Hispanic/Latino versus White	1.703	0.489	5.936	0.403
Other versus White	1.622	0.475	5.535	0.440
Sex				
Female versus Male	1.199	0.759	1.895	0.437
Education				
Bachelor’s degree or more versus Some college or less	3.746	2.384	5.887	< 0.0001

Logistic regression model examining the association between consent form version and likelihood of enrollment with demographic covariates in Experiment 2 (comparison of standard key information language vs. modified key information vs. modified key information and clarified costs)

Overall, 70% of participants gave correct responses to the question asking, “What best describes what this study is testing” (Table 4). The two forms (C and D) that had modifications to the key information section only differed by a single line stating that there were no additional costs to participate. To more easily observe the difference between the modified and unmodified forms, these two groups were combined and compared to the form without key information modifications (form B). More participants who had viewed either of the two forms with key information section modifications correctly answered, “Which of the following best describes how treatment will be decided for patients in this study?” (standard key information: 44% vs. modified key information and clarified costs forms: 52%, *p* = 0.03). Among all participants, 97% of those who answered both of these knowledge questions incorrectly were willing to enroll in the study, compared to 74% of those who answered both knowledge questions correctly (*p* < 0.0001).

## Discussion

This study was designed to assess the potential impact of changing language in two components of consent forms: (1) the description of financial implications of participation—namely, compensation for injury; and (2) the newly-required concise presentation of key information. The former (compensation for injury) is a section that is often templated and relatively “established.” It has raised concerns on the part of patients and surrogates but has never been evaluated empirically. The latter is a new requirement, and institutions and investigators have struggled to determine how it should be structured. In the context of a hypothetical study comparing two standard of care treatments in an acute care setting, modifications to both of these components of informed consent forms did not impact likelihood of enrolling a family member in the study. However, these changes did impact the understanding of some components of the study.

This study was primarily motivated by concern that off-putting and complex language that tends to characterize the compensation for injury section of most ICFs may result in potential participants declining to participate in clinical trials, particularly in trials where excess risks of injury were not of concern, because all treatments were standard of care. In the trial that inspired this study, the refusal rate was over 40%, with patients often stating concerns about financial implications of participation described in the consent form [4]. However, our experimental data do not suggest that compensation for injury concerns are a major driver of decisions since the modifications to this section did not affect willingness to enroll in the trial. Tailoring this section of the ICF did result in improved understanding of compensation for injury specific to the context of this trial (any harms would be treated just like regular medical care). In this respect, further attention to this section of ICFs in order to make it more patient-centered, clearer, and simpler may have value, even if it does not impact their ultimate decisions regarding enrollment.

Modifications to the key information page included more positively-valenced, simpler language and clearer explanations of the risks and benefits of participating in the trial example. We observed no impact of these modifications on willingness to participate. Similarly, inclusion of language regarding financial implications on the key information page had no impact on willingness to participate and did not have a significant impact on the frequency with which respondents selected concerns about costs. The only significant impact that we observed from the modifications tested in experiment 2 was on understanding of study features such as randomization. The fact that this impact was much larger in one of the novel forms and not in the other, despite no difference in content between the two novel forms related to study features, suggests that this effect is likely modest. Especially given that it is a new aspect of the ICF document, further experimentation and innovation related to the key information page is essential in order to optimize any desirable impacts this section can have on decision-making.

We did observe a negative association in both experiments between comprehension of either study features or compensation for injury and participants’ willingness to enroll. Among those who selected the ‘most wrong’ response to the compensation for injury question—that the sponsor would cover all costs—almost all were willing to enroll a family member in the study. Similarly, there was an inverse relationship between correctly answering questions about both treatment assignment and what the study was testing and willingness to enroll a family member in this study. Although these observations occurred in a hypothetical context, they are concerning and provide some support for the value of efforts to increase comprehension in designing ICFs.

An additional surprising finding was the observation that willingness to enroll a family member in the study increased significantly between the first and second experiments among individuals exposed to the same ICF (Form B: Tailored Compensation Language). In addition, the percentage of participants who answered both of the knowledge questions correctly decreased in experiment 2 compared to experiment 1, despite being exposed to this same ICF. There were differences in the characteristics of the population for the second experiment, namely experience as a surrogate, education, and race. These differences did not compromise the validity of the project because each experiment was independently randomized at the participant level, and comparison groups were well-balanced. However, the findings highlight the hazards of using historical controls and other non-randomized designs.

This study does have limitations. Perhaps most important is the hypothetical nature of the experiments. Hypothetical willingness to enroll a family member in this trial may not reflect actual willingness to enroll. In the real context for this trial, participants would have the opportunity to ask coordinators and clinicians questions about the study and would presumably have greater engagement and understanding of the condition itself. In addition, a real-life scenario would likely involve more significant pressure to make a timely treatment decision for a family member experiencing a medical emergency. Just under half of the respondents had experience making treatment decisions for someone in emergency settings. Additionally, respondents for this survey were MTurk users who may have been more familiar with online surveys or research participation. In the case of the actual study on which this hypothetical context was based, there was a 40% refusal rate among eligible patients, which is lower than what was observed here. For all of these reasons, these findings should be viewed as hypothesis-generating and as providing direction regarding potential modifications that may be productive to evaluate and consider in real-world contexts.

Additional research on the impact of modifying these components of consent forms could help to explore whether these hypothesis-generating findings appear to be correct and whether other types of modifications would be impactful. Specifically, it would be productive to evaluate the effect of more significant changes to key information language on understanding and willingness to enroll in comparative effectiveness trials. A robust evidence base could play a very important role in shaping how this new element of consent is operationalized. It could also be productive to evaluate the impact of more patient-centered, patient-driven descriptions of financial implications of study participation and of more clearly-defined plans for addressing study-associated expenses. Because cost concerns were selected by about half of the participants—regardless of consent form group—further exploration of how to address this concern is warranted.

## Conclusions

In the context of a trial comparing two standard treatments, consent form modifications that emphasized simplicity and clarity of key concepts and financial implications such as compensation for injury did not play a significant role in enrollment decisions, but they did impact understanding of the trial.

These findings provide limited support for efforts to simplify consent form language and clarify key concepts of a study to improve the ability of participants and their decision makers to understand information being presented. The modifications tested in this study were, however, modest; additional research could identify whether more pronounced and innovative changes could have a meaningful impact on either enrollment decisions or comprehension. These findings suggest a need for continued innovation and evaluation of ICFs that includes input from patient stakeholders and empirical evaluation so that ICFs can fulfill their intended function of providing the information necessary for participants to make well informed decisions about research enrollment.

## Supplementary Information

**Additional file 1.** a. Experiment 1: Compensation for Injury Language Modifications; Form A. b. Experiment 1: Compensation for Injury Language Modifications; Form B. Sample language of standard and modified compensation for injury language in the consent forms used in Experiment 1.

**Additional file 2.** a. Experiment 2: Modifying Key Concepts and Clarifying Costs; Form B. b. Experiment 2: Modifying Key Concepts and Clarifying Costs; Form C. Additional File 2c. Experiment 2: Modifying Key Concepts and Clarifying Costs; Form D. Sample language of standard and modified key information sections in the consent forms used in Experiment 2.

**Additional file 3.** Amazon mechanical Turk survey. The survey that was administered along with the consent form versions in the Amazon Mechanical Turk platform.

**Additional file 4.** Concerns about participating in the study in Experiment 1. Percentage of respondents that selected concerns about including a family member in the hypothetical study, by consent form version in Experiment 1. Participants could select up to 3 concerns.

**Additional file 5.** Concerns about participating in the study in Experiment 2. Percentage of respondents that selected concerns about including a family member in the hypothetical study, by consent form version in Experiment 2. Participants could select up to 3 concerns.

## Data Availability

The datasets used and/or analyzed during the current study are available from the corresponding author on reasonable request.

## Abbreviation

ICF: Informed consent form
