# Supplementary material for: Experimenting with modifications to consent forms in comparative effectiveness research: understanding the impact of language about financial implications and key information
Source: BMC Med Ethics. 2022 Mar 27;23:34. doi: 10.1186/s12910-021-00736-x (PMC8962560; doi:10.1186/s12910-021-00736-x)
Supplement: Supplementary file 1 — Additional file 1. a. Experiment 1: Compensation for Injury Language Modifications; Form A. b. Experiment 1: Compensation for Injury Language Modifications; Form B. Sample language of standard and modified compensation for injury language in the consent forms used in Experiment 1. [file 12910_2021_736_MOESM1_ESM.docx]

*
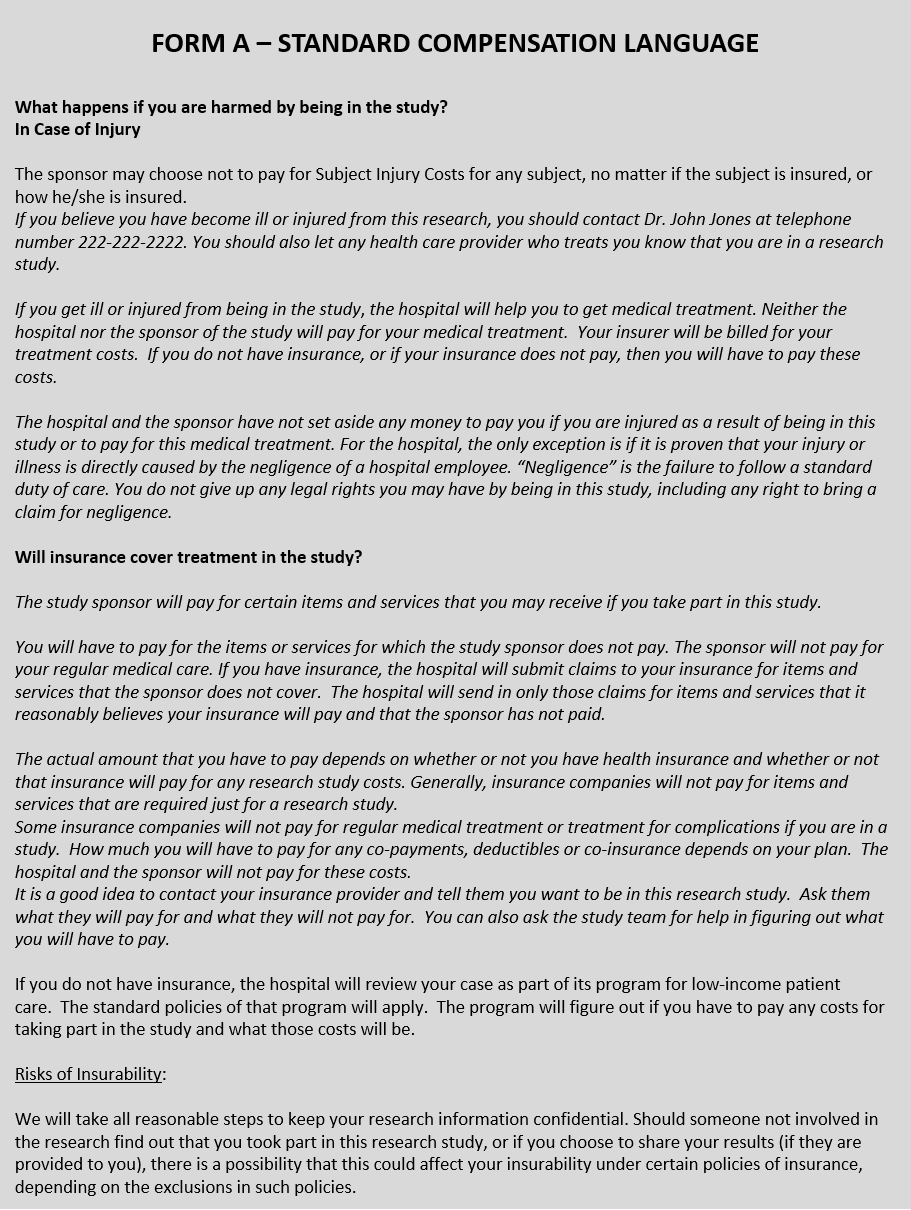
Additional File 1a. Experiment 1: Compensation for Injury Language Modifications; Form A*

*Additional File 1b. Experiment 1: Compensation for Injury Language Modifications; Form B*

*
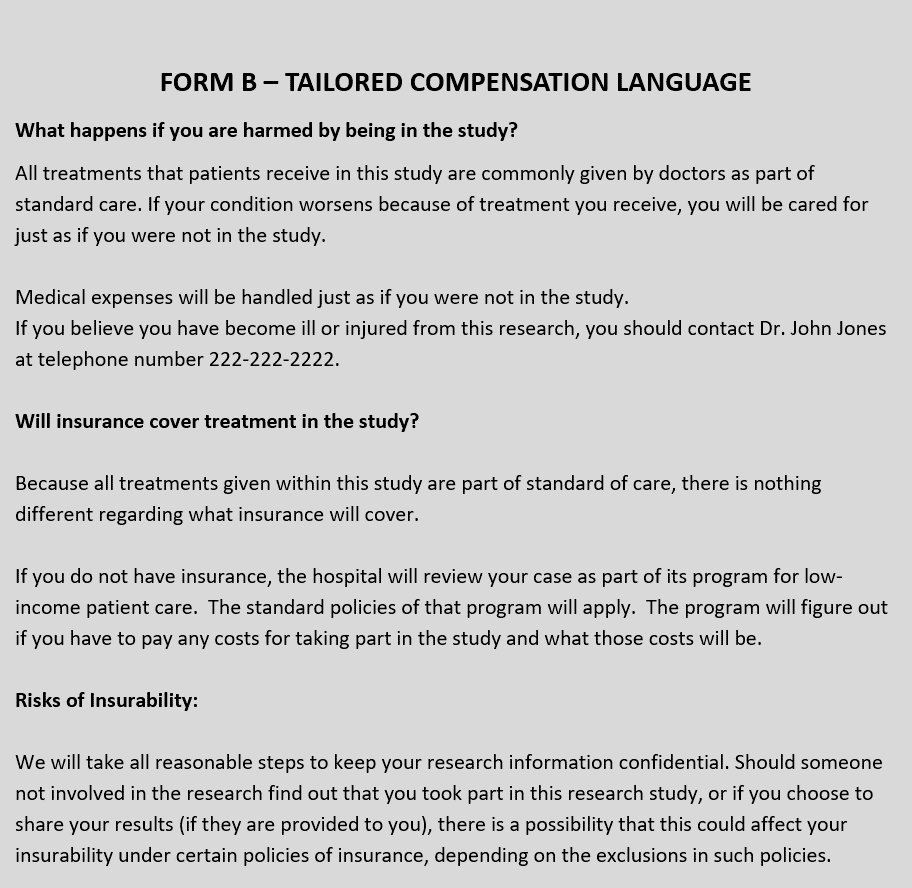
*
