# Supplementary material for: Experimenting with modifications to consent forms in comparative effectiveness research: understanding the impact of language about financial implications and key information
Source: BMC Med Ethics. 2022 Mar 27;23:34. doi: 10.1186/s12910-021-00736-x (PMC8962560; doi:10.1186/s12910-021-00736-x)
Supplement: Supplementary file 2 — Additional file 2. a. Experiment 2: Modifying Key Concepts and Clarifying Costs; Form B. b. Experiment 2: Modifying Key Concepts and Clarifying Costs; Form C. Additional File 2c. Experiment 2: Modifying Key Concepts and Clarifying Costs; Form D. Sample language of standard and modified key information sections in the consent forms used in Experiment 2. [file 12910_2021_736_MOESM2_ESM.docx]

*Additional File 2a. Experiment 2: Modifying Key Concepts and Clarifying Costs; Form B*

*
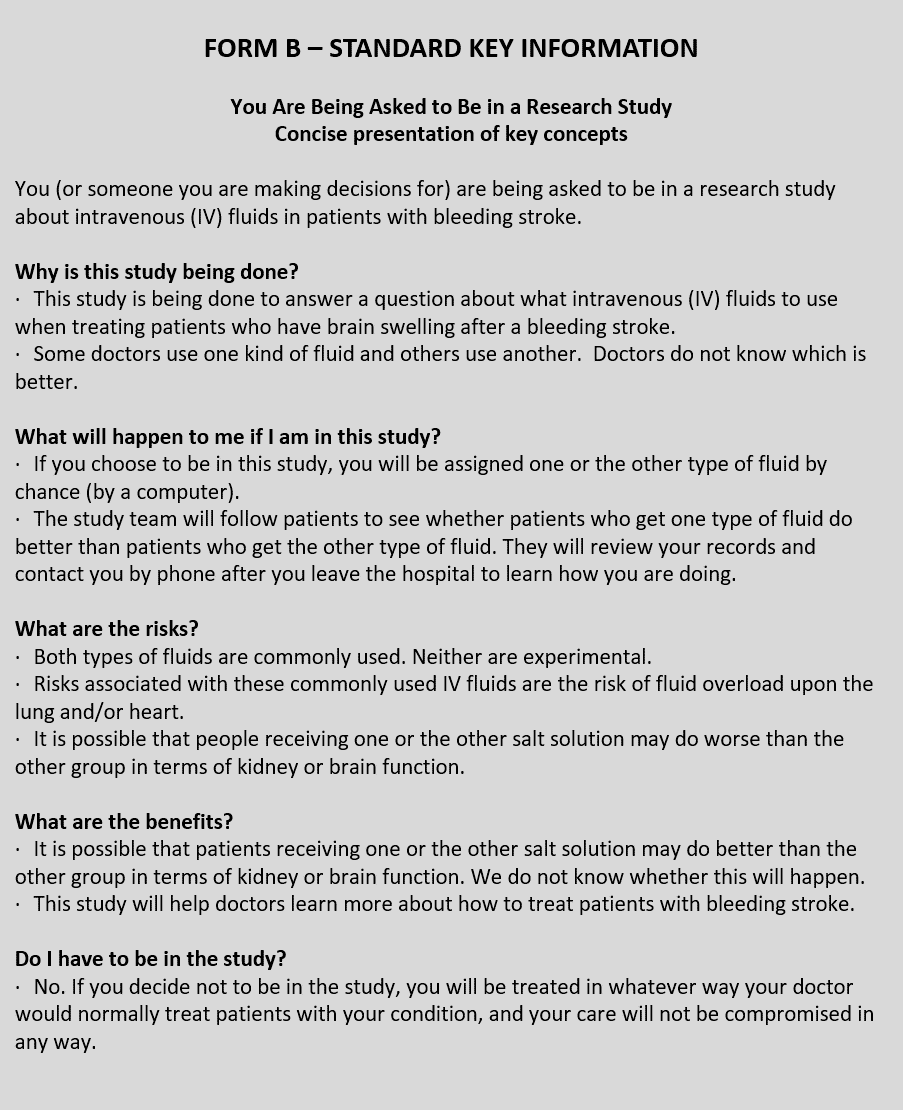
*

*Additional File 2b. Experiment 2: Modifying Key Concepts and Clarifying Costs; Form C*

*
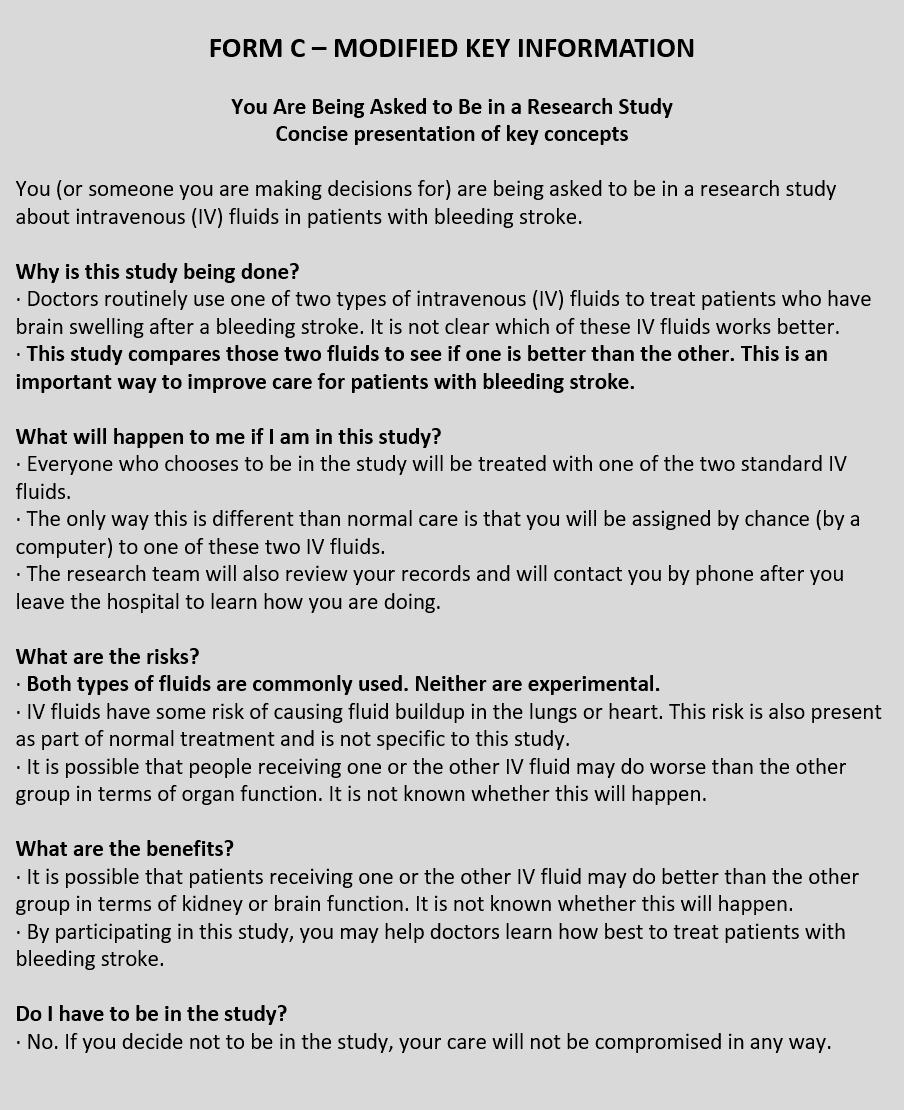
*

*Additional File 2c. Experiment 2: Modifying Key Concepts and Clarifying Costs; Form D*

*
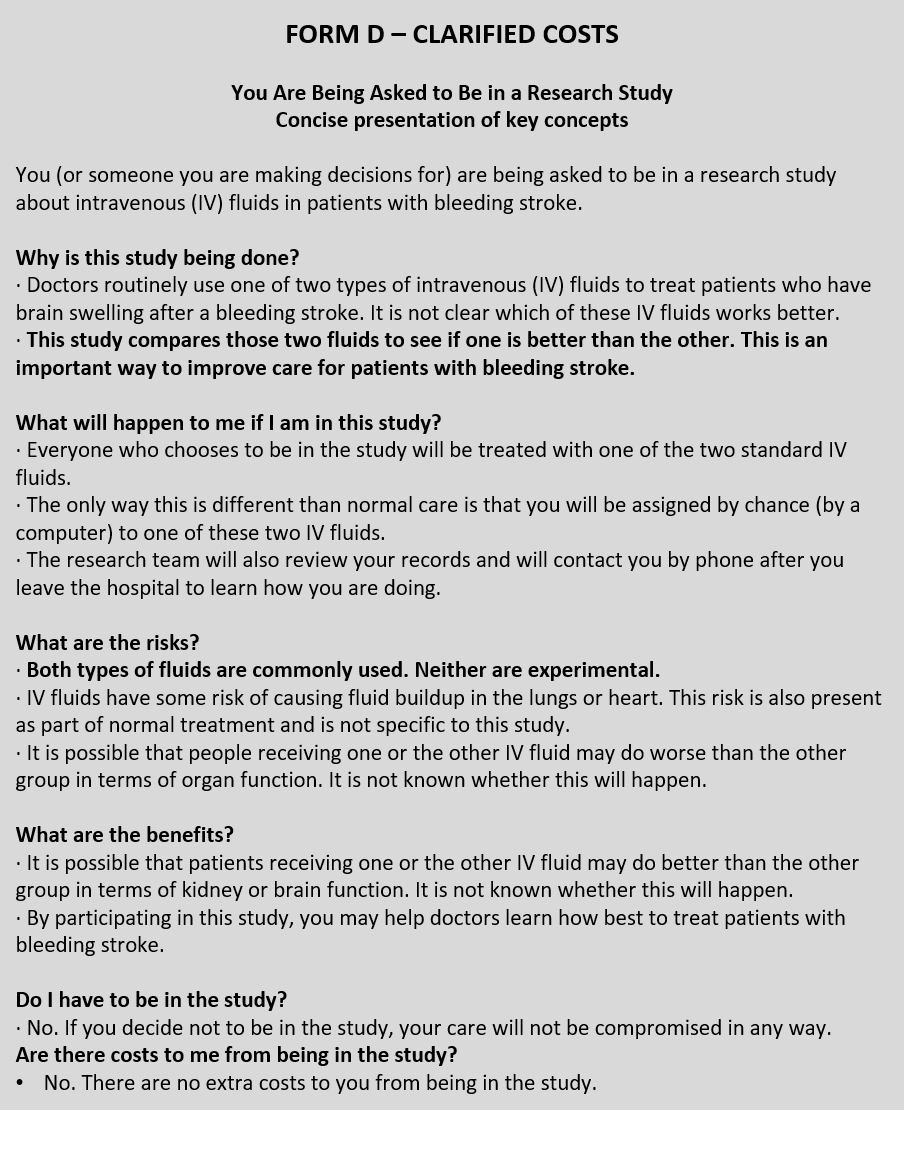
*
