# Supplementary material for: Experimenting with modifications to consent forms in comparative effectiveness research: understanding the impact of language about financial implications and key information
Source: BMC Med Ethics. 2022 Mar 27;23:34. doi: 10.1186/s12910-021-00736-x (PMC8962560; doi:10.1186/s12910-021-00736-x)
Supplement: Supplementary file 3 — Additional file 3. Amazon mechanical Turk survey. The survey that was administered along with the consent form versions in the Amazon Mechanical Turk platform. [file 12910_2021_736_MOESM3_ESM.docx]

**Additional File 3**

**Amazon Mechanical Turk Survey**

Thank you for your interest in this survey. This survey is designed to help researchers understand how people think about research that tests ways to treat medical conditions when different doctors use different approaches. This survey is being done by researchers from the Georgia Clinical and Translational Science Alliance and is funded by the National Institutes of Health. If you have any questions, please contact the Principal Investigator, Neal Dickert at 404-712-6834.

This survey will take about 15 minutes to complete. We will not collect identifiable health information about you. As with all MTurk surveys, you do not have to do the survey if you do not want to do it.  It is entirely your choice.

The purpose of this study is to learn about people’s thoughts about research studies that compare different treatment methods for specific medical conditions. In this survey, we would like you to read a consent form for a research study that involves comparing two treatments for a stroke, and then answer questions about it. For all of these questions, we would like you to imagine that your family member has suffered a stroke and you are the medical decision-maker who will determine if your family member enrolls in the study.

**[RANDOMIZE HERE]**

**Group A: Consent 1 - Original**

Please read the consent form carefully before answering the questions about it.

[_____]

**Group B: Consent 2 – Simple version**

Please read the consent form carefully before answering the questions about it.

[_____]

**Survey Questions - ALL RESPONDENTS**

Please read the following statements and choose the best answer:

1. Which of the following best describes how treatment will be decided for patients in this study?

1. A doctor will decide each patient’s treatment group based on what he/she thinks is best for that patient.
2. A computer will randomly assign each patient to one of the two treatment groups.
3. The patient’s family member will decide which treatment the patient receives.

2. What best describes what this study is testing?

1. The impact of patients’ blood types on rates of recovery from this type of stroke.
2. How well patients with this type of stroke do when treated with one type of IV fluid compared to another.
3. The frequency of different complications with this type of stroke based on where the patient is treated.

3. This type of stroke is serious and life threatening. To show that you are reading this question, please choose the answer that best describes this type of stroke. *The third answer is the correct one.*

- 1. This type of stroke is not serious.
  2. This type of stroke is not life threatening.

1. This type of stroke is serious and life threatening.

4. Again, imagine you are asked to give permission for your family member to be included in this head to head study.

After reading this consent form how likely are you to give permission to include your family member in this study?

| 1 – Very Unlikely | 2 - Unlikely | 3 - Likely | 4- Very Likely |
| --- | --- | --- | --- |
| 1 | 2 | 3 | 4 |

5. What, if any, would be your concerns about including your family member in this research study? (Choose up to 3):

1. Concerned about privacy of health information.
2. Do not trust researchers.
3. Concerned about cost of treatment or complications.
4. Concerned about risk.
5. Concerned about random assignment (by computer).
6. Concerned about getting the less effective treatment.
7. Other (please specify_____________).
8. No specific concerns.

6. If you are injured or harmed as a result of being in this study, how will your care be paid for?

1. Just like medical care outside of a research study
2. The sponsor of the study will provide it free of charge
3. Insurance companies may treat research harms or injuries differently than regular medical care
4. It may be different for every patient
5. It is not clear based on the information I read

7. On a scale of 1 to 5 with 1 being Not Confident at All and 5 being Extremely Confident, please rate how confident you are in the answer above.

| Not Confident at All |  |  |  | Extremely Confident |
| --- | --- | --- | --- | --- |
| 1 | 2 | 3 | 4 | 5 |

8. Have you ever been involved in making a treatment decision for someone who needs emergency medical care?

- 1. Yes
  2. No

9. What medical condition is this head to head study treating?

- 1. Bleeding stroke (subarachnoid hemorrhage)
  2. Congestive heart failure
  3. Kidney failure

10. What year were you born? __________

11. What is your gender?

- 1. Female
  2. Male
  3. Prefer to self-describe: ____________________
  4. Prefer not to answer

12. Which of the following category or categories best describe you (check all that apply)?

- 1. American Indian, Native American, or Alaska Native
  2. Asian
  3. Black or African American
  4. Hispanic/Latino(a)
  5. Middle Eastern or North African/Mediterranean
  6. Native Hawaiian or Pacific Islander
  7. White or European American
  8. Multi-Race – please specify:________________
  9. Other – please specify:____________________

13. What is your highest level of education?

- - - - 1. Less than high school
        2. High school graduate or GED
        3. Some college (associated, trade school, etc.)
        4. Bachelor’s degree
        5. Postgraduate (master’s doctoral) or Professional degree
