# Supplementary material for: Experimenting with modifications to consent forms in comparative effectiveness research: understanding the impact of language about financial implications and key information
Source: BMC Med Ethics. 2022 Mar 27;23:34. doi: 10.1186/s12910-021-00736-x (PMC8962560; doi:10.1186/s12910-021-00736-x)
Supplement: Supplementary file 5 — Additional file 5. Concerns about participating in the study in Experiment 2. Percentage of respondents that selected concerns about including a family member in the hypothetical study, by consent form version in Experiment 2. Participants could select up to 3 concerns. [file 12910_2021_736_MOESM5_ESM.docx]

**Additional File 5**

Concerns about participating in the study in Experiment 2

*Figure legend:* *Percentage of respondents that selected concerns about including a family member in the hypothetical study, by consent form version in Experiment 2. Participants could select up to 3 concerns.*
